# Preparation and Evaluation of Starch Hydrogel/Contact Lens Composites as Epigallocatechin Gallate Delivery Systems for Inhibition of Bacterial Adhesion

**DOI:** 10.3389/fbioe.2021.759303

**Published:** 2021-11-16

**Authors:** Lianghui Zhao, Hongwei Wang, Chengcheng Feng, Fangying Song, Xianli Du

**Affiliations:** ^1^ Qingdao Eye Hospital of Shandong First Medical University, Qingdao, China; ^2^ State Key Laboratory Cultivation Base, Shandong Provincial Key Laboratory of Ophthalmology, Shandong Eye Institute, Shandong First Medical University and Shandong Academy of Medical Sciences, Qingdao, China; ^3^ Weifang Medical University, Weifang, China

**Keywords:** starch hydrogel, composite, contact lens, epigallocatechin gallate, antibacterial activity

## Abstract

Microbial infections caused by wearing contact lenses has become a major health problem, so the design and development of antibacterial contact lenses has attracted widespread attention. To safely and effectively inhibit bacterial adhesion of contact lenses, we have facilely prepared epigallocatechin gallate (EGCG) loaded starch hydrogel/contact lens composites by *in-situ* free radical polymerization of the mixture containing 2-hydroxylethyl methacrylate, methacrylic acid and ethylene glycol dimethacrylate. The adequate transmittance of the resulting contact lenses was characterized by ultraviolet-visible spectrophotometry, and their satisfactory stability was examined using differential scanning calorimetry and thermogravimetric analysis. Whereafter, cytotoxicity and degradation experiments were performed to investigate the biocompatibility and degradability of the contact lenses. The results showed the nontoxicity and good degradability of the composites. Besides, the capacity of the contact lenses for *in vitro* release of EGCG was also evaluated, and the results showed that the EGCG in these contact lenses can be sustainably released for at least 14 days. Further bacterial adhesion assay suggested that the EGCG loaded starch hydrogel/contact lenses could significantly reduce the adhesion of *Pseudomonas aeruginosa* compared to the control. The EGCG loaded starch hydrogel/contact lens composites provide a potential intervention strategy for preventing ocular microbial infections and inhibiting bacterial keratitis.

## Introduction

There are more than 140 million contact lenses wearers all over the world (Stapleton et al., 2007). The contact lenses have been widely utilized in the vision correction of nearsightedness (myopia), far-sightedness (hyperopia), presbyopia and astigmatism. However, it can also cause several ocular discomforts, such as neovascularization, acute red eye and corneal abrasion (Kates and Tuli, 2021). More seriously, bacterial keratitis was easily induced during wearing or storage of contact lenses, especially in the case of corneal abrasion (Liu et al., 2020) due to the bacterial adhesion and biofilm formation on their surface. *Pseudomonas aeruginosa* (*P. aeruginosa*), an opportunistic Gram-negative pathogen, is the most common cause for the contact lens-associated bacterial keratitis (Hilliam et al., 2020; Spernovasilis et al., 2020). Biofilm growth of *P. aeruginosa* can enhance the tolerance to antibiotic agents and host immune responses, which involves regulation in its quorum sensing and expression of virulence genes (Liu et al., 2020). The resulting *P. aeruginosa* keratitis can lead to permanent vision loss or even eyeball removal without the prompt treatment (Vazirani et al., 2015; Fernandes et al., 2016). Therefore, it is of significance to develop the highly biocompatible and antimicrobial contact lenses for effective prevention of contact lens-associated bacterial keratitis (Seggio et al., 2019; Fleiszig et al., 2020; Meretoudi et al., 2021).

Due to the complex ocular surface structure and strong resistance to foreign body transport, the drug often stays on the ocular surface for a short time and has low bioavailability, resulting in unsatisfactory therapeutic effects (Gaudana et al., 2010). With the development of material technology, it is possible to drugs deliver to the ocular surface continuously using drug-loaded contact lenses as delivery carriers. With the limited tear exchange rate, the contact lenses can greatly increase the residence time of the drug on the ocular surface and improve the bioavailability (Xu et al., 2018).

Although drug-loaded contact lenses have broad application prospects, the safety and biocompatibility are worrisome. Edible polysaccharide starch has been widely used in the fields of pharmaceutical and biomedical engineering due to its wide sources, low cost, good biocompatibility and degradability (Pandey et al., 2015). It has been reported that the hydrophobic starch can be used as delivery carrier, and the drug can also be chemically bonded to the starch backbone for sustained release, which indicates that starch is an ideal and safe drug delivery system (Pandey et al., 2015; Kou et al., 2020).

EGCG, a member of the polyphenol family, is the main biologically active ingredient in green tea (Ouyang et al., 2020). With a wide range of biological activities including antioxidant, anti-tumor, anti-inflammatory, anti-fibrosis, anti-microbe, antivirus, anti-obesity, EGCG has been used in the prevention and treatment of bacterial infection, cancer and obesity research (Cui et al., 2012; Kwak et al., 2016; Suzuki et al., 2016; Basu et al., 2018; Crous-Masó et al., 2018). In recent years, it has been reported that EGCG can protect lens epithelial cells from UV damage, prevent tryptophan oxidation of γ-crystallin in cataract in the presence of H_2_O_2_, slow down retinal degeneration, and relieve dry eye inflammation in rabbits and mice (Heo et al., 2008; Peng et al., 2010; Lee et al., 2011; Chaudhury et al., 2015; Tseng et al., 2016; Perdices et al., 2020) due to its antioxidant, anti-inflammatory and anti-apoptotic activities. However, the antibacterial activity of EGCG has been little studied in ophthalmic drug delivery systems.

**FIGURE 1:**
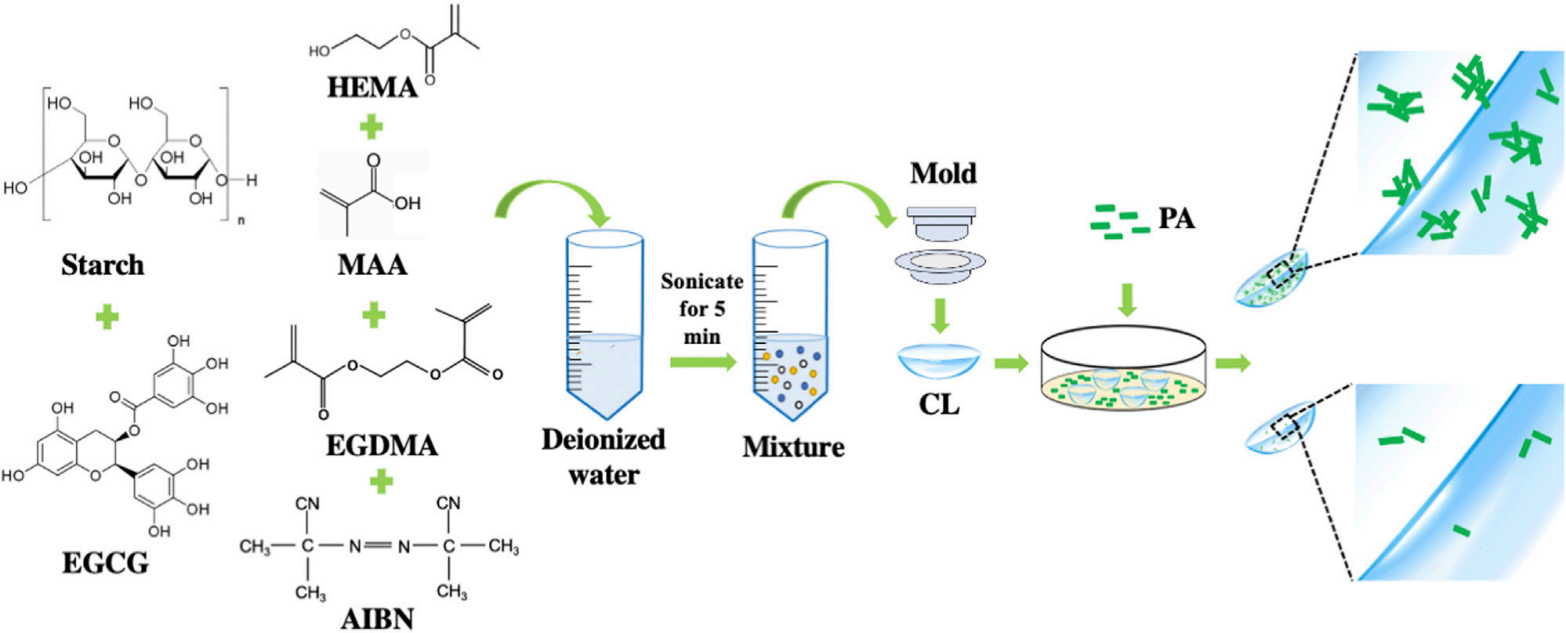
Schematic preparation of epigallocatechin gallate loaded starch hydrogel/contact lens composites.

In this study, we firstly reported the preparation of EGCG loaded starch hydrogel/contact lens composites by *in-situ* free radical polymerization of the mixture containing 2-hydroxylethyl methacrylate, methacrylic acid, ethylene glycol dimethacrylate, AIBN, starch and EGCG. The resulting composites were assessed in their physical characterizations, *in vitro* EGCG release, *in vitro* biocompatibility and *in vitro* degradability. Then bacterial adhesion inhibition effects were further evaluated by bacterial adhesion assay (Figure 1).

## Materials and Methods

### Reagents

2-Hydroxylethyl methacrylate (HEMA), *Ipomoea* batatas starch and EGCG were obtained from Macklin (Shanghai, China). Methacrylic acid (MAA) and ethylene glycol dimethacrylate (EGDMA) were obtained from ThermoFisher (United States). 2,2′-azobis (2-methylpropionitrile) (AIBN) was obtained from Sigma-Aldrich (United States) and deionized water was used in the study.

### Preparation Methods

Different amounts of starch solution (33.3%, w%), EGCG (0, 0.3, 0.6 and 1%, w%), HEMA/MAA (70:30, 70 wt%), EGDMA (0.45 wt%), and AIBN (0.15 wt%) were mixed into the deionized water. The mixture was sonicated for 5 min, transferred into the contact lens molds, placed in a water-bath at 65°C for 30 h, and then boiled for 5 min to remove the unreacted monomers, affording contact lenses CL-0, CL-1, CL-2, CL-3. Conventional hydrogel contact lenses were prepared according to the same method mentioned above except the absence of starch (CL-1′, CL-2′, CL-3’) or both starch and EGCG (CL).

### Physical Characterizations

The optical transmittances of the contact lenses were measured with an ultraviolet-visible spectrophotometer (SpectraMax M2, Molecular Devices, MD, United States) at 50 nm intervals in the wavelength range of 250–800 nm. The contact lenses were removed from the molds, dried, and the dry weight (Wd) was recorded. Then immerse the contact lenses in deionized water to assess their expansion performance. Take out the lenses at regular intervals and drain the lenses surface completely with filter paper, and record their wet weight (Wt).

Swelling ratio(%)=(Wt-Wd)/Wd

Differential scanning calorimetry (DSC) detection and thermogravimetric analysis (TGA) were performed through the synchronous thermal analyzer (TA DSC-TGA Q600, United States). The experiment was performed in the following conditions: nitrogen purity, 99.999%; temperature, 20–600°C; sample weight, 5–10 mg. Fourier transform infrared spectrum analysis, X-ray photoelectron spectroscopy, and other characterization tests were carried out for the contact lenses with or without starch.

### *In vitro* Release Study

The contact lenses were immersed into 4 ml of deionized water. At a predetermined time point, 1 ml of the release medium was taken out and replaced with an equal amount of fresh deionized water. NanoDrop One (Thermo, United States) was used to determine the content of EGCG at the wavelength of 278 nm. Then the cumulative release curve of EGCG with time was plotted by GraphPad Prism 9. The experiment was repeated three times.

### Cytotoxicity Test

The cytotoxicity of EGCG loaded starch hydrogel/contact lens composites was evaluated based on the effects of contact lenses extracts on the proliferation of human corneal epithelial cells (HCECs, Seoul, Korea) through cell counting kit-8 (CCK-8) assay. 2.5 and 5 mg of different type contact lenses were respectively dipped in 10 ml DMEM/F12 (Sigma-Aldrich) containing 10% (v/v) fetal bovine serum (Sigma-Aldrich) and 1% (v/v) antibiotic/antimycotics (Sigma-Aldrich) in 37°C, 5% CO_2_ incubator for 24 h to obtain contact lenses extracts with concentrations of 1:4 and 1:2 (Li et al., 2020). While the HCECs were plated into a 96-well plate at 2000/well and cultured in standard culture medium (DMEM/F12, 10% FBS, 1% antibiotic/antimycotics) for 24 h in 37°C, 5% CO_2_ incubator. Then, the culture medium of all the wells was taken out and different group cells were cultured with the prepared contact lens extracts along with fresh standard culture medium, respectively. After culturing in 37°C, 5% CO_2_ incubator for 1, 3, 5 and 7 days, CCK-8 detection reagent (Dojindo Laboratories Kumamoto, Japan) was added to measure the absorbance of the cells at 450 nm wavelength with SpectraMax M2 (Molecular Devices, United States).

### *In vitro* Degradation Assay

The *in vitro* degradation of starch in the EGCG loaded starch hydrogel/contact lens composites was evaluated by *Bacillus* alpha-amylase (Solarbio, Beijing, China, pH 5.5–7.5, T 50–70°C). Briefly, the contact lenses were immersed in iodine solution for 2 min, and the color of contact lenses was observed by slit lamp microscope. The contact lenses were rinsed gently with deionized water to remove residual iodine solution, and then soaked in active alpha-amylase solution for enzymatic hydrolysis at 50°C for 24 h (0.1 mg/ml and 0.01 mg/ml, 10 ml). At estimated time points, the colorless lenses were taken out and softly rinsed with deionized water to remove residual amylase solution. After addition of iodine solution, the contact lenses were further photographed to detect the color reaction of starch and iodine.

### Bacterial Adhesion Assay

*P. aeruginosa* ATCC 19660 inoculum was cultured on LB agar medium at 37°C, while planktonic culture was grown with an initial optical density (OD) of ∼0.02 and shaking in an incubator at 37°C for 24 h (Li et al., 2020). The bacteria were washed with PBS and then serially diluted to 10^6^ CFU/ml. The contact lenses were softly washed with PBS in 24 pore culture plate, and added in 1 ml prepared bacterial suspension, then incubated at 37°C and 120 rpm for 24 h. After incubation, the cocultures of the contact lenses and bacteria were photographed and the number of bacteria left on the lenses was counted. In brief, contact lenses were gently rinsed 3 times with PBS to remove loosely adhered bacteria, then grinded to detect the number of adhered bacteria by plate counting (Salvagni et al., 2020).

### Statistical Analysis

Statistical analysis was performed using Kruskal–Wallis test with Statistical Package for Social Sciences software (version 24.0). All the data were obtained from at least three independent experiments. The descriptive statistics were presented as the mean ± SD. *p* < 0.05 was considered a statistically significant difference.

## Results and Discussion

Microbial infection is a common problem associated with contact lenses, and poor sanitary conditions and overnight wearing contact lenses are the main risk factors for microbial keratitis (Cope et al., 2015; Cope et al., 2017). Long-term contact between cornea and contact lenses will affect the nutrient exchange of normal corneal epithelial cells, causing ocular discomfort or serious complications (Forte et al., 2010; Lim et al., 2018). Hypoxia environment of ocular surface can destruct the integrity of extracellular matrix proteins, promote bacterial adhesion, and provide a good condition for opportunistic pathogens to invade the cornea (Forte et al., 2010). In recent years, with the development of drug delivery, the design and exploitation of antimicrobial contact lenses had attracted extensive attention.

*In-situ* free radical polymerization is a common method for preparation of therapeutic contact lenses by addition of bioactive ingredients. In this study, antibacterial contact lenses were facilely prepared by thermally initiated polymerization of the mixture containing starch, EGCG and conventional hydrogel components. Starch significantly improves the sustained release performance of conventional hydrogel contact lenses without changing the basic physical properties.

### Synthesis and Characterizations of the Contact Lenses

The contact lenses were prepared according to specific ratio of different ingredients as reported previously (Dursch et al., 2014). In short, different amounts of EGCG (0, 0.3, 0.6 and 1%, w/w), hydrogel solution and AIBN were added to the starch solution and prepared into CL-0, CL-1, CL-2, CL-3 using specific contact lenses models, while CL was prepared as negative control.

**FIGURE 2:**
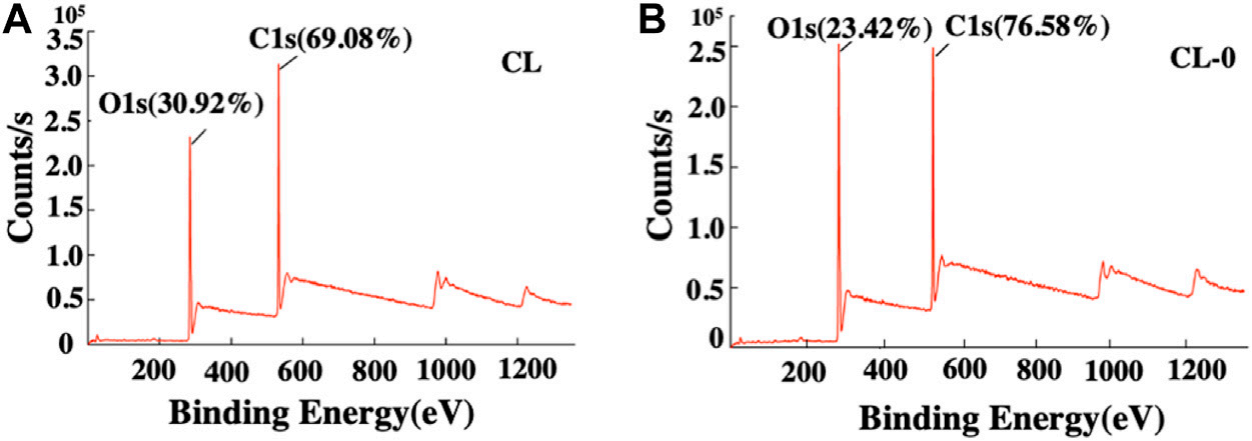
Elemental analysis of the contact lenses CL **(A)** and CL-0 **(B)**.

The addition of starch was confirmed through element analysis as shown in Figure 2. The initial carbon and oxygen content in CL was 69.08 and 30.92%, respectively. After adding starch, the carbon content increased to 76.58% and the oxygen content reduced to 23.42%, indicating the successful preparation of starch hydrogel/contact lenses. Studies have confirmed that the natural polymers are non-toxic and biodegradable and can bind non-specifically or specifically to proteins and cells, which are the ideal materials for hydrogel-based sustained-release systems or cellular carriers (Kirchhof et al., 2015). As a kind of natural polysaccharide with wide sources and good biocompatibility, starch is widely used in pharmaceutical and biomedical engineering fields, which was selected as the component of the contact lenses in present study (Pandey et al., 2015; Hadisi et al., 2018; Kou et al., 2020). Based on our data, the addition of starch to the hydrogel material does not affect the basic properties of contact lenses such as transparency, optical transmittance, swelling, thermal stability and thermoplasticity.

**FIGURE 3:**
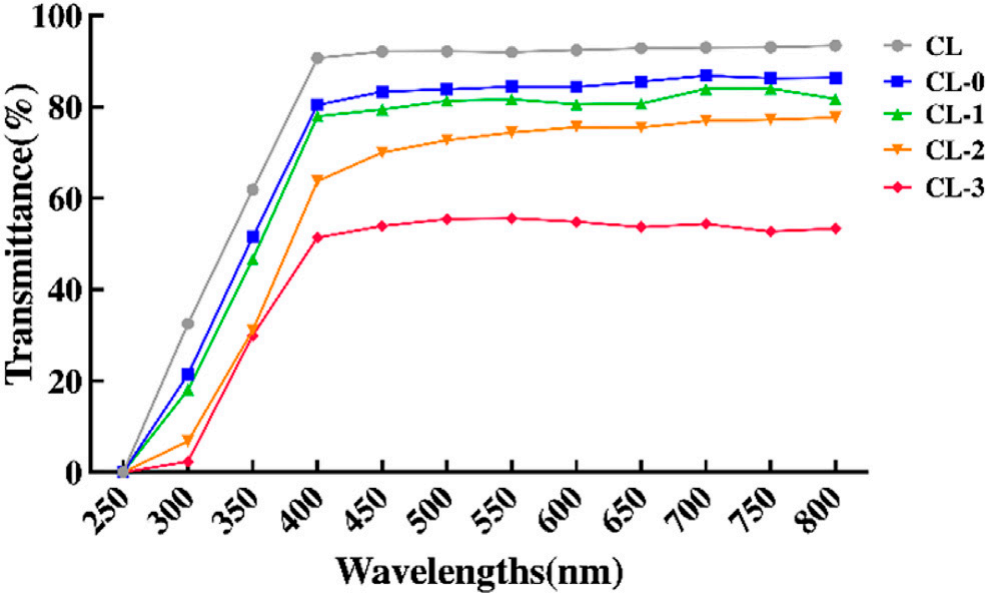
Optical transmittance of the contact lenses.

**FIGURE 4:**
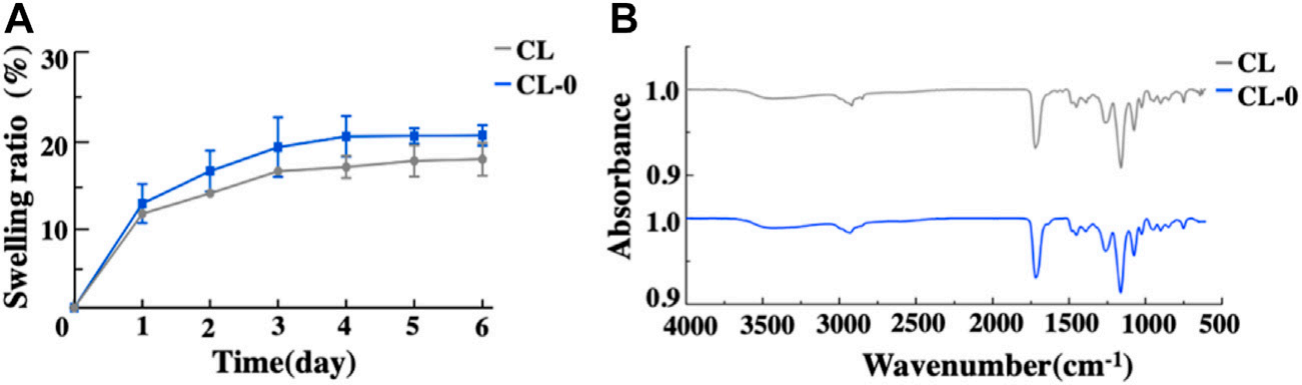
Swelling ratio **(A)** and Fourier transform infrared spectroscopy analysis of the contact lenses **(B)**.

The optical transmittance data of contact lenses with different treatments are shown in Figure 3. Compared with the negative control CL, there was no significant difference in transmittance between CL-0 and CL group, while the optical transmittance of starch hydrogel/contact lenses (CL-0) decreased slightly (Figure 3), indicating that the addition of starch had little effect on transmittance. In EGCG group, with the addition of EGCG, the color of contact lenses became yellowish and the optical transmittance gradually decreased (CL-1, 84.00 ± 0.72%; CL-2, 77.70 ± 0.90%; CL-3, 54.37 ± 0.53%; Supplementary Figure S1, Figure 3). Furthermore, swelling test results confirmed that the swelling rate of CL-0 group (21.92 ± 1.30%) was higher than that of CL group (18.88 ± 2.10%) after immersion in deionized water for 24 h, (Figure 4A). It was indicated that starch improved the swelling ratio of contact lenses to a certain extent.

**FIGURE 5:**
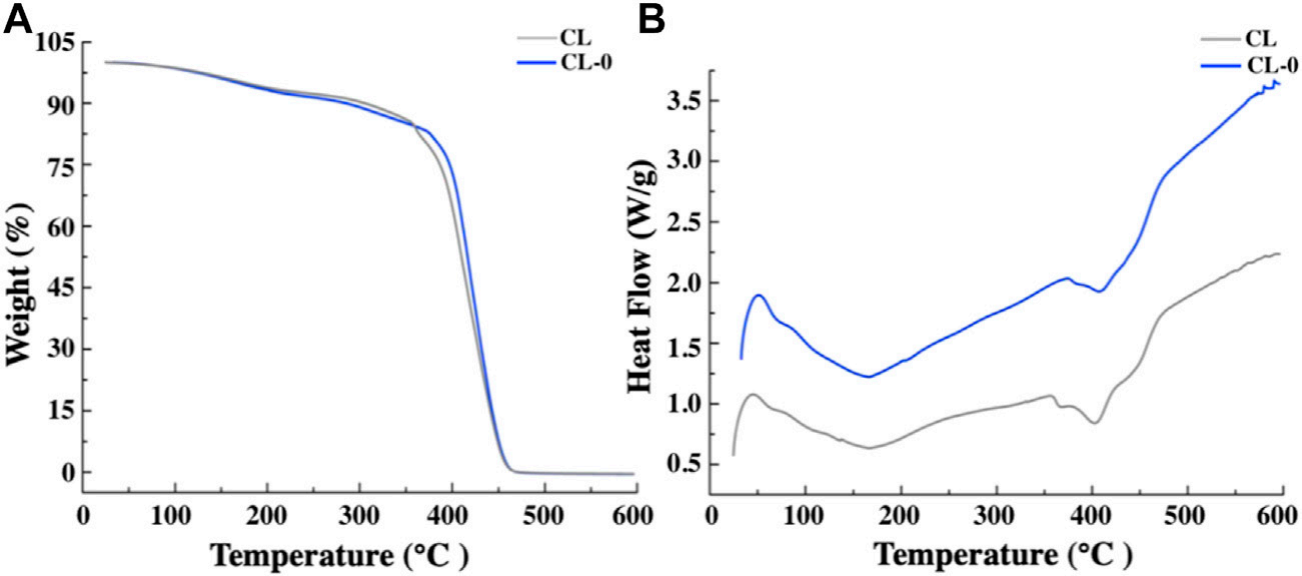
Thermogravimetric analysis **(A)** and Differential scanning calorimetry test of the contact lenses **(B)**.

Fourier transform infrared spectroscopy analysis showed that the corresponding functional groups of starch hydrogel contact lens did not change significantly. In addition, the characteristic peak of starch infrared absorption spectrum didn’t appear due to the low starch content (Figure 4B). Figure 5A showed the similar TGA curves of CL and CL-0 groups. It was clearly found that both samples could maintain 90% of weight even the temperature rose to 300°C. Furthermore, the DSC melting curve of CL group was similar as that of CL-0. The addition of starch has no significant effect on the material fusibility (Figure 5B). These results confirmed the good thermal stability and thermoplasticity of EGCG loaded starch hydrogel/contact lens composites.

### Biocompatibility

**FIGURE 6:**
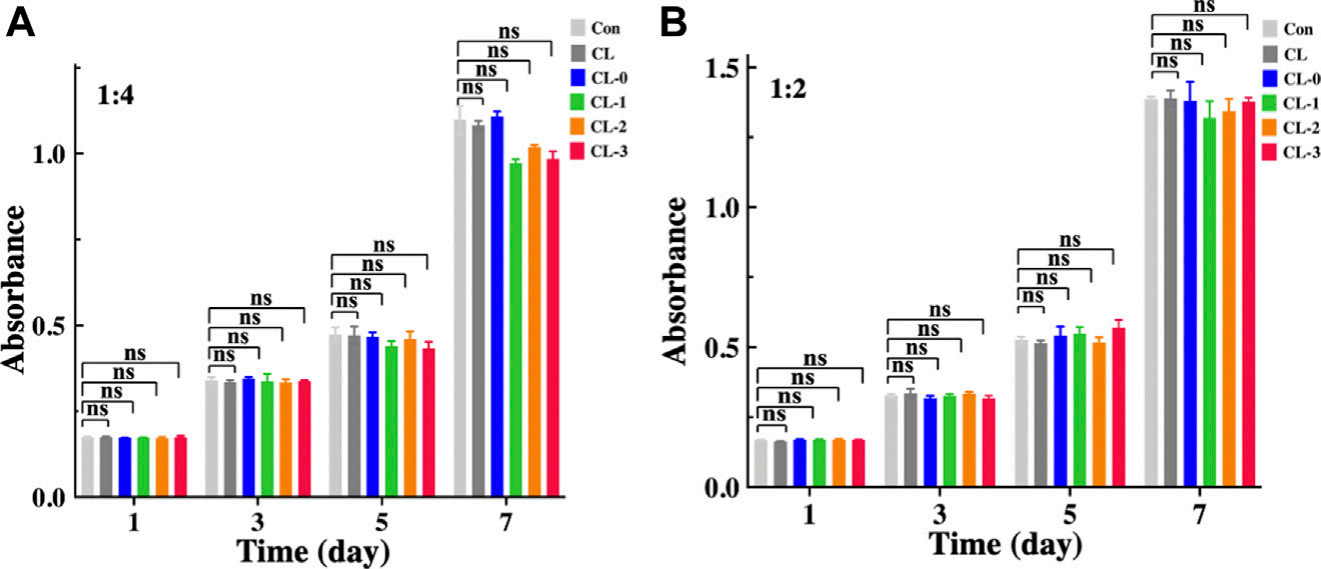
Cytotoxicity and proliferation activities of the contact lenses on human corneal epithelial cells (HCECs). Cell counting kit-8 analysis of HCECs seeded in the contact lenses extracts with a concentration of 1:4 **(A)** and 1:2 **(B)**, *n* = 3.

Biosafety is a great challenge in the development of functional contact lenses. Table 1 lists the research representatives on antimicrobial contact lenses recently, showing that a variety of antimicrobial components are loaded on different types of contact lenses. These contact lenses mainly bind variable antibacterial materials to carriers such as hydrogels through *in-situ* synthesis, coating or covalently attachment. Although functional contact lenses containing antibiotics, non-steroidal anti-inflammatory drugs or silver ions have significant antibacterial properties, they could cause certain adverse reactions, such as the emergence of drug-resistant strains, eye irritation, and biocompatibility problems of metal ions (Silva et al., 2018; Xiao et al., 2018; Hoyo et al., 2019; Nahum et al., 2019; Khan and Lee, 2020; Salvagni et al., 2020). In this study, cytotoxicity test was investigated to evaluate the cytocompatibility of EGCG-loaded starch hydrogel/contact lenses. After different types of contact lenses were immersed in standard medium for 24 h, the cytotoxicity of contact lenses extracts against HCECs were detected. Based on absorbance data of the solution at 450 nm wavelength, it was showed that contact lens CL-0, CL-1, CL-2 and CL-3 extracts had no inhibitory effect on the proliferation of HCECs compared with untreated HCECs and CL group (Figure 6). There was no significant difference in the absorbance between ECGC groups. Those results demonstrated that EGCG loaded starch hydrogel/contact lens composite exhibited no obvious cytotoxicity, which makes it possible to be applied in the medical field in the future.

**TABLE 1:** Representatives of antimicrobial contact lenses in recent years.

Substrates	Antimicrobial agents	Release time(d)	Biological activity	References
*In-situ* synthesis				
Starch/poly (HEMA-co-MAA-co-EGDMA)	EGCG	14	Antibacterial	In this study
Poly (HTCC-co-HEMA)	HTCC, Vor, Ag	5	Antifungal	Huang et al. (2016)
Silicone/poly (TRIS-co-HEMA-co-NVP-EGDMA)	ALG, CHI, HA, PLL	2–5	Antibacterial	Silva et al. (2018)
Poly (HEMA-co-MAA-co-EGDMA)	Silver NPs	——	Antibacterial	Shayani Rad et al. (2016)
Commercial CL				
Hioxifilcon A	AMPs	7	Antibacterial	Salvagni et al. (2020)
Narafilcon A	Ofloxacin, vitamin E	4.28	Antibacterial	Ubani-Ukoma et al. (2019)
Acuvue	Phomopsidione NPs	4	Antibacterial	Bin Sahadan et al. (2019)
Silicone hydrogel	Vancomycin	0.33	Antibacterial	Guo et al. (2020)
Comfilcon A	ZnO, chitosan, gallic acid	——	Antibacterial	Hoyo et al. (2019)
Narafilcon A	Esculentin-1a (1-21)NH_2_	——	Antibacterial	Casciaro et al. (2017)

EGCG, epigallocatechin gallate; HTCC, N-[(2-Hydroxy-3-trimethylammonium) Propyl] Chitosan Chloride; Vor, voriconazole; TRIS, 3-tris (trimethylsilyloxy)silylpropyl 2-methylprop-2-enoate; NVP, N- vinyl pyrrolidone; ALG, sodium alginate; CHI, chitosan; HA, sodium hyaluronate; PLL, polylysine hydrobromide; NPs, nanoparticles; CL, contact lens; AMPs, antimicrobial peptides.

### *In vitro* Degradation by Alpha-Amylase

**FIGURE 7:**
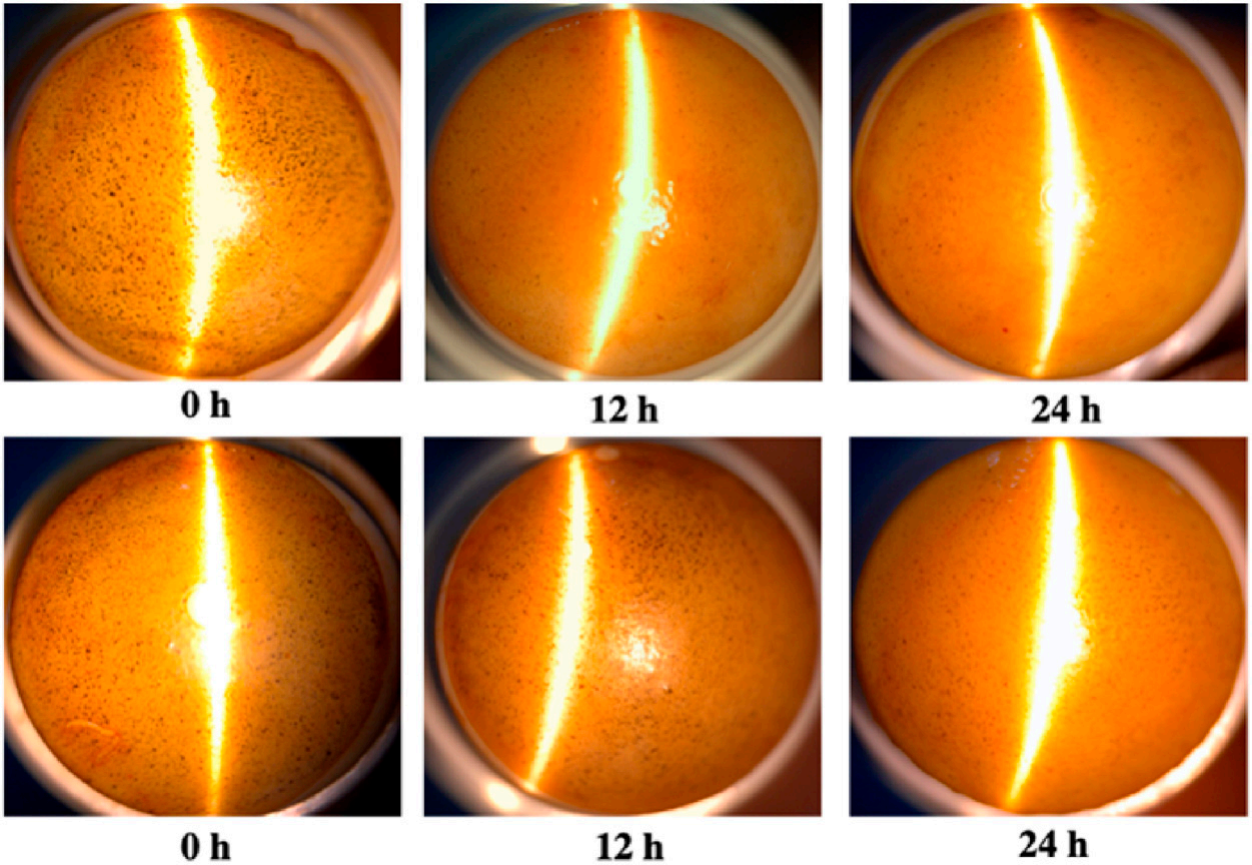
*In vitro* degradation of epigallocatechin gallate loaded starch hydrogel/contact lens composites by alpha-amylase. The concentration of active alpha-amylase was 0.1 mg/ml **(A)** and 0.01 mg/ml **(B)**.

In order to evaluate the degradability of materials, an *in vitro* alpha-amylase degradability detection of the composites was performed. The EGCG starch hydrogel/contact lens composite (CL-1) was stained with iodine solution and showed dark blue spots on the surface. After incubation with active alpha-amylase solution, the amounts of blue spots on the surface decreased with the increase of enzymatic hydrolysis time. The blue spots on the lenses disappeared after 12 h of enzymatic hydrolysis using the 0.1 mg/ml alpha-amylase solution, while there were still a few blue spots on the lenses after 24 h of enzymatic hydrolysis using the 0.01 mg/ml alpha-amylase solution (Figure 7). Above all, it was suggested that the EGCG starch hydrogel/contact lens composites had good degradability.

### *In vitro* EGCG Release

**FIGURE 8:**
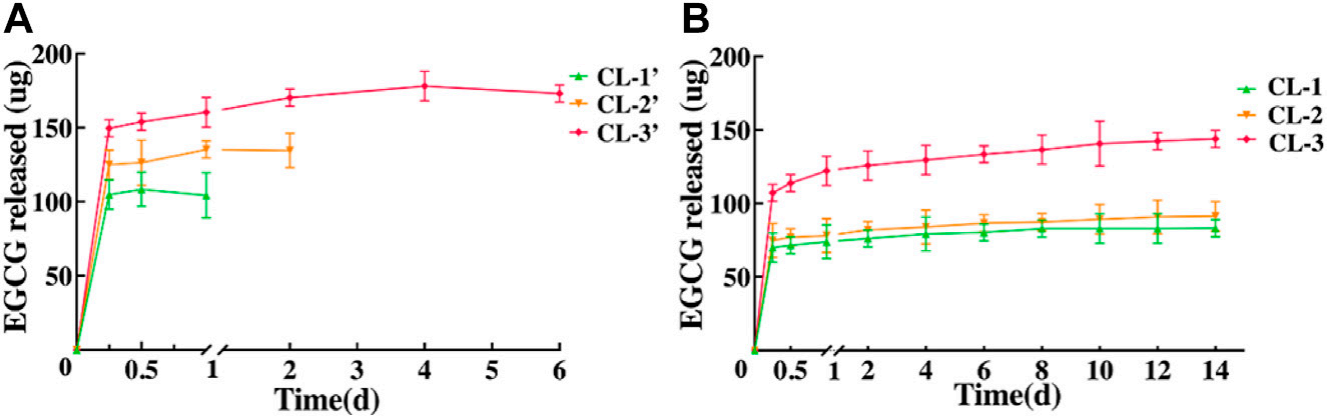
*In vitro* EGCG release from conventional hydrogel contact lenses **(A)** and starch hydrogel/contact lenses **(B)**.

EGCG is the main biologically active ingredient in tea, which has a wide range of antioxidant, anti-tumor, anti-inflammatory, anti-fibrosis, anti-microbe, antivirus, anti-obesity and other activities (Cui et al., 2012; Kwak et al., 2016; Suzuki et al., 2016; Basu et al., 2018; Crous-Masó et al., 2018; Ouyang et al., 2020). In view of the safety and eligible biocompatibility of contact lens extracts against HCECs, we further tested the EGCG release of those contact lenses. After being immersed in deionized water, the cumulative release amount of EGCG from the EGCG loaded starch hydrogel/contact lenses was detected at different time points (Figure 8). The results exhibited that the EGCG in these contact lenses (CL-1, CL-2, CL-3) can be sustainably released for at least 14 days. The durations are obviously longer than those (less than 4 days) in conventional hydrogel contact lenses (Figure 8A). With the increase of EGCG amount in contact lenses, the cumulative release of EGCG continuously increased from 82.79 ± 5.77 to 143.97 ± 5.77 ug (Figure 8B).

It has been reported that drug delivery systems with good sustained release properties, such as peptide-functionalized contact lenses, have a sustained-release time of approximately 7 days (Salvagni et al., 2020). In this study, the sustained-release capacity of EGCG loaded starch hydrogel/contact lens composites were greatly improved. It was indicated that the properties of drug delivery of the contact lenses in this study were effectively improved compared with the commercial CL (Table 1). Extending the drug release time of lenses plays an important role in the long-term inhibition of bacterial adhesion, which is beneficial for prolonging the safe wearing time of contact lenses in the future.

### Antimicrobial Activity

Many *in vitro* studies have shown that *P. aeruginosa* had the strongest adhesion to silicone hydrogel and hydrogel contact lenses, reaching the maximum adhesion of bacterial within 1 h, and forming biofilm within 24 h (Dutta et al., 2012). The formation of biofilms relies on the bacterial quorum sensing system to sense and regulate the diffusion signal molecules related to the bacterial population density and the expression of virulence factors, thus providing an ecosystem for bacteria to enhance their virulence and drug resistance (Jimenez et al., 2012).

**FIGURE 9:**
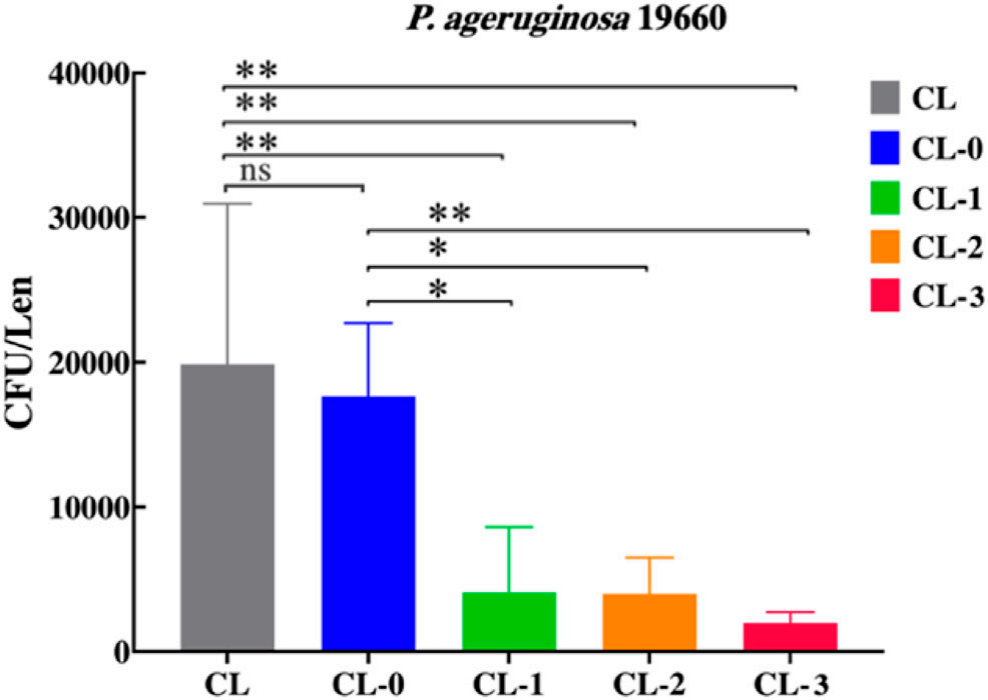
Adhesion of *P. aeruginosa* on the contact lenses determined by viable plate count.

To examine the antimicrobial activity of those contact lenses, bacterial adhesion assay against *P. aeruginosa* ATCC 19660 was performed. After 24 h of incubation with *P. aeruginosa*, the amounts of bacteria adhered onto lenses were quantified. Compared with CL and CL-0, the coculture liquid of CL-1, CL-2 and CL-3 was more clearly and the amount of bacterial adhesion was significantly reduced (*p* < 0.05) (Supplementary Figure S2). Compared with CL and CL-0, the amounts of bacterial adhesion on CL-1, CL-2, CL-3 are significantly reduced (*p* < 0.05). With the increase of EGCG content, the amount of bacterial adhesion on contact lenses decreased gradually (CL-1, 4531 ± 4100; CL-2, 3967 ± 2517; CL-3, 1967 ± 751) (Figure 9). These results indicated that the composites could effectively inhibit the adhesion of *P. aeruginosa*, which provides the possibility to reduce the incidence of contact lens induced *P. aeruginosa* keratitis in the future.

## Conclusion

In summary, the EGCG loaded starch hydrogel/contact lens composites have been successfully prepared by *in-situ* free radical polymerization of the mixture containing HEMA, MAA, EGDMA, starch and EGCG. The resulting composites exhibited favorable biocompatibility and superior drug release ability for EGCG at least 14 days, which processed enhanced sustained release capacity of EGCG than conventional hydrogel contact lens (4 days). Hence, they can effectively inhibit the adhesion of *P. aeruginosa* on the lenses, which is beneficial for prolonging the safe wearing of contact lenses and provides the potential for the clinical therapy of bacterial keratitis in the future. Furthermore, the study enriches the research scope of functional contact lenses, and various natural products with specific activities can be introduced in the preparation of therapeutic contact lenses for treatment of other ophthalmological diseases.

## Data Availability

The original contributions presented in the study are included in the article/Supplementary Material, further inquiries can be directed to the corresponding authors.
