# Supplementary material for: Preparation and Evaluation of Starch Hydrogel/Contact Lens Composites as Epigallocatechin Gallate Delivery Systems for Inhibition of Bacterial Adhesion
Source: Front Bioeng Biotechnol. 2021 Nov 16;9:759303. doi: 10.3389/fbioe.2021.759303 (PMC8637123; doi:10.3389/fbioe.2021.759303)

**Preparation and evaluation of starch hydrogel/contact lens** **composites as epigallocatechin gallate delivery systems for inhibition of bacterial adhesion**

Lianghui Zhao^1,2,3^, Hongwei Wang^1,2^, Chengcheng Feng^1,2^, Fangying Song^1,2*^, Xianli Du^1,2*^

^1^Qingdao Eye Hospital of Shandong First Medical University, Qingdao, Shandong 266071, China.

^2^State Key Laboratory Cultivation Base, Shandong Provincial Key Laboratory of Ophthalmology, Shandong Eye Institute, Shandong First Medical University & Shandong Academy of Medical Sciences, Qingdao, Shandong 266071, China.

^3^Weifang Medical University, Weifang, Shandong 261021, China.

* Correspondence authors:

Fangying Song (E-mail: songfangying_@126.com, Tel.: +86-532-85881625);

Xianli Du (E-mail: lilibestever@126.com, Tel.: +86-532-85881625).

**Supplementary Figure S1**

The transparency of contact lenses


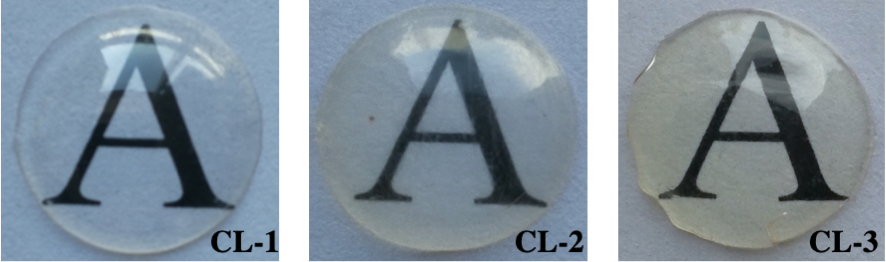


**Supplementary Figure S2**

The photos after the coculture between composites and *P. aeruginosa*.


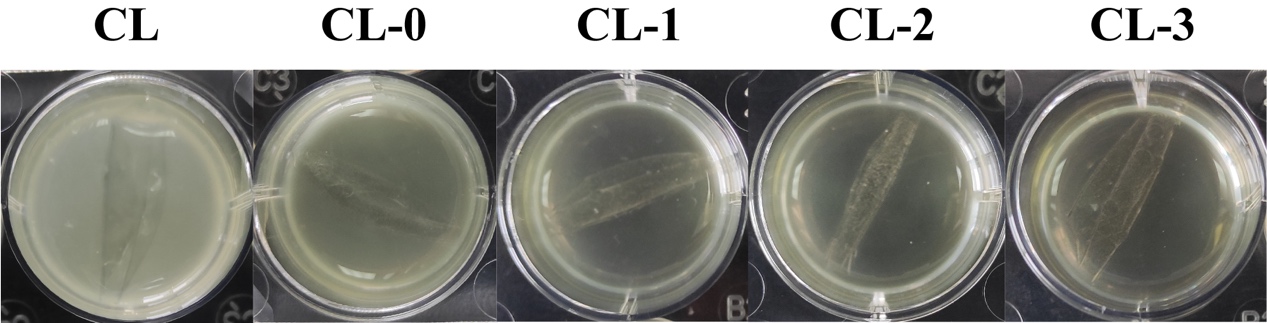

Supplement: Supplementary file 1 [file DataSheet1.docx]
